# Improving access to emergent spinal care through knowledge translation: an ethnographic study

**DOI:** 10.1186/1472-6963-14-169

**Published:** 2014-04-14

**Authors:** Fiona Webster, Michael G Fehlings, Kathleen Rice, Harsha Malempati, Khaled Fawaz, Fred Nicholls, Navindra Baldeo, Scott Reeves, Anoushka Singh, Henry Ahn, Howard Ginsberg, Albert J Yee

**Affiliations:** 1 Department of Family & Community Medicine, University of Toronto, 500 University Ave, 5th floor, Toronto, Ontario M5G 1 V7, Canada; 2 Division of Neurosurgery, Department of Surgery, University of Toronto, Toronto, ON. Toronto Western Hospital, 399 Bathurst St, Toronto, Ontario M5T 2S8, Canada; 3 Department of Anthropology, University of Toronto, 19 Russell Street, Toronto, Ontario M5S 2S2, Canada; 4 Division of Orthopaedic Surgery, Sunnybrook Health Sciences Centre, 2075 Bayview Ave, Toronto, Ontario M4N 3 M5, Canada; 5 Orthopaedic Surgery, Cairo University, Cairo, Egypt; 6 Department of Surgery, University of Toronto, 149 College Street, Toronto, Ontario M5T 1P5, Canada; 7 Institute of Health Policy, Management and Evaluation, University of Toronto, 155 College Street, 4th floor, M5T 3 M6 Toronto, Ontario, Canada; 8 Center for Innovation in Interprofessional Education, University of California, 530 Parnassus Avenue, Library, San Francisco, CA 94143, USA; 9 Krembil Neurosciences Department, Toronto Western Hospital University Health Network, 399 Bathurst St, Toronto, Ontario M5T 2S8, Canada; 10 Division of Orthopaedic Surgery, Department of Surgery, University of Toronto, 149 College Street, Toronto, Ontario M5T 1P5, Canada; 11 Division of Neurosurgery, Department of Surgery, University of Toronto, Toronto, ON. St. Michael’s Hospital, 30 Bond St., 3 Bond Wing, Toronto, ON M5B 1 W8, Canada; 12 Division of Orthopaedic Surgery, Department of Surgery, University of Toronto, 149 College Street, Toronto, Ontario M5T 1P5, Canada

**Keywords:** Spine care, Coordination of care, Competing priorities, Ethnography, Trauma knowledge translation

## Abstract

**Background:**

For patients and family members, access to timely specialty medical care for emergent spinal conditions is a significant stressor to an already serious condition. Timing to surgical care for emergent spinal conditions such as spinal trauma is an important predictor of outcome. However, few studies have explored ethnographically the views of surgeons and other key stakeholders on issues related to patient access and care for emergent spine conditions. The primary study objective was to determine the challenges to the provision of timely care as well as to identify areas of opportunities to enhance care delivery.

**Methods:**

An ethnographic study of key administrative and clinical care providers involved in the triage and care of patients referred through CritiCall Ontario was undertaken utilizing standard methods of qualitative inquiry. This comprised 21 interviews with people involved in varying capacities with the provision of emergent spinal care, as well as qualitative observations on an orthopaedic/neurosurgical ward, in operating theatres, and at CritiCall Ontario’s call centre.

**Results:**

Several themes were identified and organized into categories that range from inter-professional collaboration through to issues of hospital-level resources and the role of relationships between hospitals and external organizations at the provincial level. Underlying many of these issues is the nature of the medically complex emergent spine patient and the scientific evidentiary base upon which best practice care is delivered. Through the implementation of knowledge translation strategies facilitated from this research, a reduction of patient transfers out of province was observed in the one-year period following program implementation.

**Conclusions:**

Our findings suggest that competing priorities at both the hospital and provincial level create challenges in the delivery of spinal care. Key stakeholders recognized spinal care as aligning with multiple priorities such as emergent/critical care, medical through surgical, acute through rehabilitative, disease-based (i.e. trauma, cancer), and wait times initiatives. However, despite newly implemented strategies, there continues to be increasing trends over time in the number of spinal CritiCall Ontario referrals. This reinforces the need for ongoing inter-professional efforts in care delivery that take into account the institutional contexts that may constrain individual or team efforts.

## Background

Delays in access to timely specialty medical care for emergent spinal conditions are a significant stressor for patients and family members. Timing to surgical care for emergent spinal conditions such as spinal trauma and spinal cord injury is an important predictor of outcome [1]. In the province of Ontario, the majority of care for acute surgical spinal conditions, including both traumatic and non-traumatic causes, is delivered by spine specialists practicing in Academic Health Science Centres (AHSCs). Emergent referrals are often made to these AHSC spinal centres through CritiCall Ontario, an integrated provincial communication and triage program. There is an ongoing mandate among key stakeholders involved in the provision of care to enhance the coordination of clinical care for these patients.

**Figure 1:**
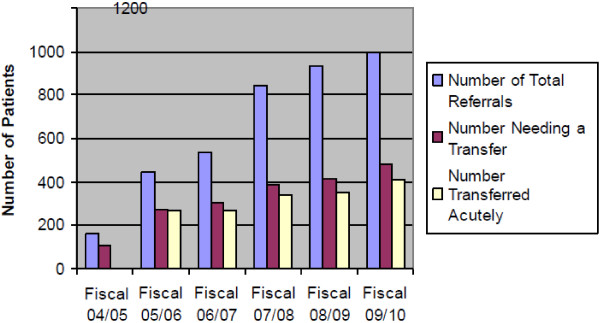
Overall spinal referrals to CritiCall Ontario 2004 and 2011.

While the need for better access to musculoskeletal surgical procedures such as total joint replacement surgery and hip fracture is well documented in Ontario [2-6], less has been reported on access and care for emergent conditions of the spine [7-9]. A preliminary pilot audit of adult spine subspecialty provincial data (CritiCall Ontario, fiscal years 2004 through 2009) was performed by two members of the research team (AJY, MGF). Based on this review, the number of patients that required an emergent transfer from a peripheral referring hospital to a specialized spinal centre was observed to have increased significantly over time. In addition, the number of overall spinal referrals to CritiCall Ontario has increased five to six-fold between 2004 and 2011 (Figure 1). This presents an important ongoing challenge to the coordination and delivery of care in the province. Delays at any point across the care continuum can adversely affect clinical outcomes [10,11].

Access to timely care remains a challenge in the current Canadian health care environment [1,3,5,6]. Emergent spinal surgery is important to patient satisfaction, quality of life, and functional outcome for conditions including acute cauda equina syndrome, spinal infection and trauma with progressive neurologic deficits [1,9]. A recent challenge in Ontario has been the timely transfer of patients from peripheral referral hospitals to spinal AHSCs for emergent care. Increases in emergent out of province transfers motivated the present research (Table 1). Mindful of the importance of timely surgery for optimal patient outcome and awareness of the importance of providing better access to surgery in Ontario, the primary objective of the study was to identify challenges as well as areas of opportunity for implementation of knowledge translation strategies. A second objective was to determine the potential impact of key knowledge translation strategies derived from collaborative input of stakeholders on the transfer of patients.

**Table 1:** Out of province acute transfers (number of patients)

Fiscal 04/05	Fiscal 05/06	Fiscal 06/07	Fiscal 07/08	Fiscal 08/09	Fiscal 09/10	Oct 2010- Sept 2011*
0	1	14	20	34	31	5

*One year data (October 2010 through September 2011) following implementation of knowledge translation strategies.

An ethnographic approach was considered ideal for our study as ethnography is the study of behavior in its naturally occurring context. As such, ethnography goes beyond traditional individual-level explanations that currently permeate the knowledge translation literature [12]. Through this approach the cultural norms, local context and specific needs of various professions can be explicated when building an account of how policymakers, clinicians, and hospital administrators interact. This understanding is a key initial step to the ultimate goal of utilizing knowledge translation strategies to enhance spinal care delivery in the health care system.

Qualitative research is inductive and does not begin with a hypothesis to be tested but instead begins by identifying an area to be explored. The primary research question we addressed was: what are the existing barriers to and opportunities to improve the implementation of emergent spine care in Ontario? Building on the findings from this study we hoped to develop knowledge translation strategies to address these barriers. The RATS guidelines for reporting qualitative research was used to ensure quality in the reporting of our study in relation to sampling, recruitment, role of researchers, ethics, analysis and discussion [13].

## Methods

### Study design and population

Following appropriate institutional research ethics board (REB) approval through the Sunnybrook Health Sciences REB Committee, an ethnographic study was conducted to explore the experiences of care providers in both academic and community settings, policymakers, and hospital administrators’ in relation to the provision of emergent spine care service. In this paper we report on the ethnographic findings of our study which included key informants involved in the triage and care of patients referred through CritiCall Ontario. Our approach was influenced theoretically by the work of sociologist ethnographer Dorothy Smith, particularly her emphasis on how the social organization of knowledge allows for an examination of the complex social relations organizing people’s experiences of their everyday working lives and how this work is coordinated with others [14-16]. For Smith, people’s everyday lives can be studied as sites of interface between individuals and a vast network of institutional relations, discourses, and work processes. Our participants included: patient flow personnel (managers, service providers) in Academic Health Science Centres, referring primary care and specialist physicians, nurses, and technical teams in Northern, rural and AHSC settings and provincial agency representatives (CritiCall Ontario, Local Health Integration Network (LHIN)).

### Sampling, recruitment, interviewing, and observations

An experienced qualitative interviewer (KR) conducted observations and face-to-face interviews with a purposive sample [17] of key stakeholders involved in making and receiving referrals for care of patients with emergent spinal conditions. Participants were identified by the research team through selection from a CritiCall distribution list of those involved in this care pathway and invited to participate in a semi-structured interview to describe their work and experiences. Written consent was obtained before the commencement of audiotaping interviews. This methodology was used to locate a range of perspectives, often referred to as maximum variation sampling in other qualitative approaches [18]. Interviews were conducted until saturation was reached and each interview was audio recorded, transcribed and entered into a qualitative software program (NVivo). Saturation refers to the point at which the interview team agrees that no new information is being produced through the interviewing process [19]. The team determined that we had reached theoretical saturation at 18 interviews and conducted three more interviews to confirm this assessment. The interviewer took care to engage the informants in a discussion that extended beyond their institutional rationale and asked participants to provide concrete examples of their work practices [20].

In addition to formal, semi-structured interviews, ethnographic observations were conducted at CritiCall Ontario’s call centre, in operating theatres and on the orthopedic surgical ward of a trauma hospital. Observations at CritiCall Ontario were deemed essential by the team since CritiCall Ontario personnel facilitate conversations between medical personnel and specialist surgeons at hospitals all over Ontario. Most decisions about where patients will be sent for care, as well as who will treat them, are made through this forum. Moreover, the challenges encountered by CritiCall Ontario personnel in their attempts to find suitable bed-space and medical care for spine patients are indicative of the limitations and pressures on the healthcare system in relation to acute spine care. Observations were carried out in the operating theatres and orthopaedic/neurosurgical surgical wards in order to gain a holistic understanding of the scope of practice, and of the workplace pressures that come to bear on patient care. The importance of understanding the surgeons’ scope of practice was identified by the surgeons themselves, many of whom felt that it would be impossible to fully grasp the state of acute spine care in Ontario without understanding their experiences in the workplace.

Prior to the observations, an email letter was sent to all staff informing them of the nature of the study and providing details as to when observations would occur. Everyone who might be observed was invited to indicate if they were uncomfortable with the observations, either before, during or following the observations. Additional verbal consent was obtained prior to each data collection period. The Hawthorne effect [21], wherein those being observed alter their behavior due to the researcher’s presence, was mitigated by several factors. While the possibility of the Hawthorne effect was discussed at every debriefing, the trauma hospital is such a busy clinical setting that the observer went relatively unnoticed. Busy teaching hospitals are also full of residents, medical students and student nurses; the data-collector (KR) was approximately the same age as these medical learners and therefore blended in well with her surroundings. Scratch notes [22] that recorded on-the-spot observations were taken and written up into detailed field-notes following these observations. All observations in the hospital took place during daytime hours, while observation at CritiCall Ontario took place overnight, since the majority of urgent calls come at nighttime hours. Between calls, informal group interviews and informal, research-related conversations were conducted with CritiCall Ontario staff.

### Data analysis

Data collection and analysis were undertaken in an iterative fashion throughout the research process; data was transcribed and coded concurrently with interviewing to allow for refining of the interview guide. At least two members of the research team (FW, KR) read transcriptions of the first two interviews independently to identify codes. The researchers then met to compare their independent analyses and a framework was developed to code the remaining transcripts. The primary author (FW) debriefed regularly with the interviewer (KR) to determine when saturation had been reached [19]. After all interviews and field notes had been coded, the larger research team met several times to identify similarities and differences across the data (FW, AY, KR). We combined our codes into themes, identified predominant ones and summarized relationships between these themes.

## Results

The 21 participants involved in this study occupied varying levels and/or roles in relation to the provision of emergent spinal care. These included orthopedic surgeons, neurosurgeons, administrators, pre-hospital staff, and other clinicians (e.g. nurses and anesthesiologists). There was unanimous agreement among providers regarding the importance of enhancing the delivery of emergent spinal care. Participants felt that there was tremendous opportunity to improve the delivery of care, by identifying barriers and developing strategies to improve health care collaboration within the system. Furthermore, they believed that this would translate into reduced health care related costs, lessen wait time to surgery, and decrease emotional pressure on patients and their families.

Several themes emerged from our analysis. We have organized our results around the tensions that arose at the professional, hospital and provincial levels and significantly impacted individual clinicians. Underlying many of these issues is the nature of the medically complex emergent spine patient and the scientific evidence outlining delivery of best practices.

### Complex patients and conflicting professional priorities

Patients with emergent spinal conditions are often medically complex. Thus, many participants spoke of a need for greater coordination between all the players. A critical care specialist, in describing the complexity of these patients, explained that many physicians were involved in their care. He said,

A lot of them, as a trauma, they’ll have polytrauma, they have other things, so spine could be the main injury but still they may have other [significant] injuries that have to be looked after … you know, it’s never one doctor …. (Health professional group, respondent 8)

In addition, it should be emphasized that trauma patients account for about 50% of critical referrals with the other 50% including degenerative, cancer and infection. In addition, these patients are often elderly and may have several comorbidities (e.g. diabetes, hypertension, cardiac disease, obesity, etc.) which further complicate the coordination of their care delivery amongst several professional specialties.

It emerged from both interviews and observations that the medical professionals who care for these patients often do so while juggling multiple priorities. These at-times-conflicting priorities are sometimes reinforced and reproduced through hospital and professional policies:

“You see, when we are on the spine call I’m not covering the spine alone. That’s a difference. As a spine specialist, you often also cover both neurosurgical or orthopaedic as well as the spine call” (Health professional group, respondent 5)

In addressing the complexity of these patients, it was apparent that caring for this population involves a great deal of inter-professional collaboration (IPC), thus further reinforcing the importance of IPC for optimal spine care:

“So that means that once the patient may be accepted by the neurosurgeon or the orthopaedic surgeon from a [spinal] surgical perspective, they still have to be accepted by the intensivist . . . because the [surgeon] can’t accept them if they need to go to an ICU [that function on a ‘closed’ unit delivery model]. So those are some of the nuances” (Health administration group, respondent 15)

### Conflicting hospital and provincial priorities

Several potentially conflicting priorities were identified by participants at the hospital and provincial level that posed a barrier to optimal care delivery. Hospital capacity, including access to in-patient hospital beds, was considered the most important potential structural barrier that would impact in the provision of timely access to care. It was recognized by many that funding for beds is linked to availability and hospitals may have competing priorities in this regard. For example, a polytrauma patient or cancer patient may be a hospital strategic priority and more likely get access to limited beds when compared to a spinal patient at a particular centre. Differential access to hospital-based resources was considered an important variable in providing timely access to care. In particular, participants regarded the widely acknowledged lack of acute care and intensive care unit (ICU) beds in hospitals as an organizational feature of care that impeded timely transfer of patients:

“We need more ICU beds. To me that [remains] a major bottleneck.” (Health professional group, respondent 10)

Also at the level of the hospital, participants recognized that spinal patients, due to their medical complexity, often require a specialized and monitored clinical care environment. This may be more difficult to coordinate from an emergent perspective. Some spinal experts spoke of the need for a specialized spine unit, referencing the example of stroke units, for post-acute and/or surgical care of these complex patients. They believed that the presence of a spine unit might improve patient flow through the health care system.

At the provincial level, spinal care specialists as well as other providers emphasized that varying level of priorities for spinal injury relative to other specialties raised challenges for them in the current care delivery environment. As one noted,

“The government has [targeted funding] for hip and knee [joint replacements] … [there is the desire] for the spinal patients populations to have the same priorities as given to the hips and knees.” (Health professional group, respondent 18)

For many, the decision to prioritize one patient group over another is based on political rather than medical or scientific evidence and several identified this as problematic. As one participant commented, *“Where they [direct their resources] is a political decision”. (Health professional group, respondent 4).* Yet despite the tensions that arose at the institutional level, the concept of interpersonal communication was frequently used to explain ongoing challenges in inter-professional collaboration. For example as one administrator told us, “*The communication with the neurosurgeons … is probably the biggest barrier. And that’s why I’m saying neurosurgeons, for spine, you can insert “orthopaedic surgeons” every time I’ve said “neurosurgeons”, okay?” (Health administration group, respondent 1).*

Finally, lack of coordination across the care continuum from acute to chronic rehabilitative and community re-integration was also identified as an area of opportunity for future change. For many clinicians and other professionals we interviewed, improving emergent spinal care cannot be focused just on issues of access for acute conditions, but must also take into account the logistics of care across the care continuum.

### Improving triage and care coordination

There was general agreement among participants that provincial infrastructure, such as CritiCall Ontario, is an essential service. Nevertheless, interviewees identified opportunities to enhance patient triage through the CritiCall Ontario system. For these specialists, time spent through the current telephone triage system and in coordinating a transfer was an added stressor to care provision. This view was also mirrored by CritiCall Ontario administrative triage staff, who described being frustrated by the amount of time spent reaching specialists.

The need for more scientific evidence in relation to spinal clearance was identified as a factor that contributed in part to delays in clearance. In addition, the use of electronic imaging systems, transparency, and linkages between care providers, hospitals, and the province were considered desirable:

“One of the barriers was we don’t know [over the phone] what you’re describing as a fracture, or what kind of fracture it is … But … if we can look at it [together electronically], then we can make a better decision.” (Health professional group, respondent 11)

There was some discussion relating to aspects of physician remuneration as it relates to patient medical complexity. Care providers recognized a varying spectrum of remuneration models and suggested that compensation for patient medical complexity could be further refined and considered in the current fee-for-service system. It was also suggested that the process of patient triage, referral, and transfer coordination involves multiple tasks and time. There may be a disincentive to care providers when considering the coordination of patients referred from outside their primary institution compared to similar patients presenting initially at their own emergency room. It is important to note that no one we interviewed suggested that they have ever refused care to a patient because of the amount of time involved in coordinating care from a referral originating outside of their primary institution.

## Discussion

Our findings illustrate that emergent care is a crucial area for inter-professional education (IPE) and IPC, as collaborative care across specialties is such a vital component of providing care for these complex patients. The importance of IPE and IPC opportunities in health care is recognized in the literature [23-25]. Arguably, the emergent spinal care patient’s medical care is particularly contingent on effective IPC. The acute nature of spinal injury and the importance of timely yet complex treatment for the well-being and quality of life of these patients compound the importance of providing such care.

Our study identified institutional-level factors that need to be addressed in order to facilitate improved access to care for emergent spine patients. Specifically strategies need to be developed that recognize the limited ability of individual physicians from different specialties to coordinate care seamlessly given constraints at the professional, hospital and provincial levels. Potential tensions between professional groups that arise as a result of organizational-level differences in responsibilities, targeted funding and resources are often masked by an emphasis on the individual interpersonal aspects of IPC, such as inter-personal communication, at the individual level [12]. Our results suggest that competing priorities at the professional, hospital and provincial levels contribute, in part, to challenges in the delivery of spinal care, given the wide spectrum of specialties involved. Spinal care coordination takes place in the context of other multiple priorities such as emergent/critical care, medical through surgical, acute through rehabilitative, disease-based (i.e. trauma, cancer), as well as wait times initiatives. Therefore the need to balance priorities in scheduled versus emergent delivery in patients that potentially require surgery is an important issue that requires a system-level response to resolve. There was a divergence of opinions regarding responsibility for emergent spinal care delivery, despite a shared vision between key stakeholders that improvements in care delivery was considered essential.

There are several important policy and practice implications that result from our findings. For example, the thematic results of this study were debriefed to key stakeholder study participants and the broader community through presentations at local and national scientific meetings and during a key panel discussion at the November, 2010 Innovation Fund Provincial Oversight Committee (IFPOC) Meeting. This meeting involved medical professional, hospital administrative, as well as provincial Ministry of Health and Long-Term Care (MOHLTC) participants. Spinal AHSCs were able to improve linkages through the provincial MOHLTC Neurosurgical expert panel responsible for enhancing access to emergent spinal care. Medical professionals and hospital administrators at spinal AHSCs worked together with the MOHLTC through the Toronto Neurosurgery Emergency Task Force Committee to address hospital resource challenges and to leverage funding gained by anticipated reductions in out of province patient transfers. AHSC and MOHLTC accountability agreements and enhanced evaluation of patient triage through CritiCall Ontario ensured transparency of key deliverables. CritiCall Ontario implemented a new Emergency Neurosurgery Image Transfer System (ENITS) permitting access to the consulting spinal specialist to computed tomography imaging performed at referring community hospitals. Recognition of the medical coordination of care through CritiCall Ontario by both referring as well as consulting physicians included a new telephone consultation fee in the Ontario Health Insurance Plan (OHIP) Schedule of Benefits. Through IPC derived efforts, a hospital and provincial based accountability agreement was implemented, including a rotating ‘last on-call rota’ physician/hospital based system, also known as a round of on-call duties.

### Limitations

There were several limitations with this study. While we attempted to capture as many perspectives as possible, the full range of experience was unlikely to have been represented. Focusing on IPC and the coordination of care delivery from the health system perspective in this study, other areas that merit evaluation includes study of patient perspectives. Results of this study may also not be generalizable to other provinces recognizing variations in regional health care models that exist in Canada. We also recognize the ongoing need to consider the continuum of care from emergent through chronic rehabilitative and community re-integration. Future work should consider IPC opportunities across the continuum of care.

## Conclusions

The product of the ethnographic phase was a rich description of how the process of coordinating referrals across sites is enacted under varying conditions of personnel, technology and availability of services. The need for empirical evidence regarding practice at the local level is important to better understand the role of context in the organization, and to facilitate uptake of best practices in health care delivery [26]. This study also stands as an excellent example of multidisciplinary research and the potential for critical qualitative research findings to have a direct impact on clinical care delivery and health care policy [27]. Enhanced IPC in the coordination of emergent care improves access to care and builds upon a shared vision of responsibility to the patient synergizing the efforts from the medical, professional, hospital administrative, through regional and provincial governance. Despite improvements in the coordination of care, there remains an opportunity to develop additional strategies for the delivery of emergent spinal care. Following the implementation of knowledge translation strategies facilitated from this research, a reduction of patient transfers out of province was observed in the one-year period following program implementation (Table 1). We also note that despite newly implemented strategies, there continues to be increasing trends over time in the number of spinal CritiCall Ontario referrals. This underscores the need for ongoing inter-professional efforts that recognize the impact and constraints of systems issues in the organization of care delivery that cannot be resolved at the level of individuals or teams.

## Competing interests

The authors declare that there are no conflicts of interests.

## Authors’ contributions

FW, MGF, AY conceived the study, and participated in its design, coordination and analysis. KR conducted the interviews and observations and contributed to analysis. FW drafted the manuscript. KR, HM, FN, NB, SR, AS, HA, HG participated in the study design and analysis. All authors read and approved the final manuscript.

## Pre-publication history

The pre-publication history for this paper can be accessed here:

http://www.biomedcentral.com/1472-6963/14/169/prepub
